# Discovery of herpesviruses in multi-infected primates using locked nucleic acids (LNA) and a bigenic PCR approach

**DOI:** 10.1186/1743-422X-4-84

**Published:** 2007-09-06

**Authors:** Sandra Prepens, Karl-Anton Kreuzer, Fabian Leendertz, Andreas Nitsche, Bernhard Ehlers

**Affiliations:** 1 P14 Molekulare Genetik und Epidemiologie von Herpesviren, Robert Koch-Institut, Nordufer 20, 13353 Berlin, Germany; 2 Klinik I für Innere Medizin, Joseph-Stelzmann-Straße 9, 50924 Köln, Germany; 3 Zentrum für Biologische Sicherheit, Robert Koch-Institut, Nordufer 20, 13353 Berlin, Germany; 4 Max-Planck-Institut für Evolutionäre Anthropologie, Deutscher Platz 6, 04103 Leipzig, Germany

## Abstract

Targeting the highly conserved herpes DNA polymerase (DPOL) gene with PCR using panherpes degenerate primers is a powerful tool to universally detect unknown herpesviruses. However, vertebrate hosts are often infected with more than one herpesvirus in the same tissue, and pan-herpes DPOL PCR often favors the amplification of one viral sequence at the expense of the others. Here we present two different technical approaches that overcome this obstacle: (i) Pan-herpes DPOL PCR is carried out in the presence of an oligonucleotide substituted with locked nucleic acids (LNA).This suppresses the amplification of a specific herpesvirus DPOL sequence by a factor of approximately 1000, thereby enabling the amplification of a second, different DPOL sequence. (ii) The less conserved glycoprotein B (gB) gene is targeted with several sets of degenerate primers that are restricted to gB genes of different herpesvirus subfamilies or genera. These techniques enable the amplification of gB and DPOL sequences of multiple viruses from a single specimen. The partial gB and DPOL sequences can be connected by long-distance PCR, producing final contiguous sequences of approximately 3.5 kbp. Such sequences include parts of two genes and therefore allow for a robust phylogenetic analysis. To illustrate this principle, six novel herpesviruses of the genera *Rhadinovirus, Lymphocryptovirus *and *Cytomegalovirus *were discovered in multi-infected samples of non-human primates and phylogenetically characterized.

## Background

PCR-based methods have been used for over a decade to discover unknown herpesviruses. VanDevanter and coworkers [1] were the first to design degenerate primers against the highly conserved DPOL gene in order to detect unknown herpesviruses by PCR. Since then, several variations of the original method were published, for example PCR based on deoxyinosine substituted primers [2] or consensus-degenerate hybrid oligonucleotide primers [3]. Despite of the tremendous efficiency of these methods in detecting previously unknown viruses [4-8], they all have a limitation: In specimens from a multi-infected individual, they usually amplify a viral sequence from only one of the herpesviruses present. For example, pigs are infected with three different lymphotropic herpesviruses (PLHV-1, PLHV-2 and PLHV-3) with high prevalence, and a considerable percentage is double- or triple- infected [9,10]. We easily detected PLHV-1 and PLHV-2 with panherpes DPOL PCR [11] but we needed another 2 years and a large collection of porcine blood and tissue samples to find PLHV-3 with the same method in a small number of PLHV-1- and PLHV-2-negative samples [9]. Retrospective analysis of the sample collection with PLHV-3-specific primers revealed that PLHV-3 was not less prevalent than PLHV-1. However, less efficient amplification of PLHV-3 by pan-herpes DPOL PCR prevented its detection in double- or triple-infected samples [unpublished data].

Another shortcoming limitation of this technique is, that the amplified sequences are short (usually <0.5 kb). Although this is beneficial for the sensitivity of the PCR, short sequences are often not sufficient for the construction of phylogenetic trees revealing acceptable probabilities for all clades.

Here we present a combination of two experimental approaches to overcome these shortcomings: (i) Pan-herpes DPOL PCR was carried out in the presence of an additional oligonucleotide modified by the introduction of locked nucleic acids (LNA). (ii) The less conserved glycoprotein B (gB) gene was amplified with degenerate primers of limited detection capacity i.e. genus-specific primers.

LNAs are ribonucleotides containing a methylene bridge that connects the 2'-oxygen of the ribose with the 4'-carbon. The result is a locked 3'- endo conformation that reduces the conformational flexibility of the ribose and forces the conformational transition from the B-type to the A-type [12]. The introduction of LNAs into DNA and RNA improves the hybridization affinity and increases the melting temperature by 1°-8°C/LNA nucleotide [13]. LNAs have been widely used for the control of gene expression, in particular for therapeutic purposes [Reviewed by: [14]]. A recent report described the use of LNAs in cDNA-based real-time PCR in order to inhibit the amplification of contaminating genomic DNA [15]. In the present study, LNAs were used for the first time to exclusively inhibit the amplification of known herpesvirus sequences, thereby facilitating the amplification of additional unknown herpesvirus sequences from multi-infected specimens.

The glycoprotein B (gB) gene is located immediately upstream of the DPOL gene in beta- and gammaherpesviruses, and is less conserved than the DPOL gene. It only allows for the design of more restricted degenerate primers i.e. gB sequences of a single herpesvirus subfamily or genus can be amplified, while sequences of viruses belonging to other genera remain excluded.

By combining these two experimental procedures, six novel primate herpesviruses of the genera *Rhadinovirus, Lymphocryptovirus *and *Cytomegalovirus *were discovered in multi-infected specimens. To determine which gB and which DPOL sequences originated from the same virus genome, the putative gB/DPOL pairs were connected by long-distance (LD) PCR. Final contiguous sequences of approximately 3.5 kbp were compiled and used for robust phylogenetic analysis.

## Methods

### Sample collection and DNA preparation

Blood and tissue samples from chimpanzees (*Pan troglodytes verus*), deceased from various reasons, were collected in the Taï National Park of Côte d'Ivoire. Samples of other Old World primates, deceased in captivity, were collected in the German Primate Centre (DPZ) and in the Zoological Gardens of Berlin, Germany (Table 1). DNA was prepared as described previously [16].

**Table 1:** Origin of samples

Primate species		Freeranging	Individuals	Organ	Origin	
					Country	Location
*Pan troglodytes verus*	Chimpanzee	+	3	Spleen, muscle	Côte d'Ivoire	Taï National Park
*Papio hamadryas*	Hamadryas baboon	-	5	Lung, spleen, liver, lymph node, heart	Germany	German Primate Center
*Macaca fascicularis*	Cynomolgus monkey	-	4	Blood, spleen, oesophagus	Germany	German Primate Center
*Colobus guereza*	Black-and-white colobus	-	4	Blood, liver, oral mucosa	Germany	Zoological Gardens, Berlin

### Pan-herpes PCR with specificity for the DNA polymerase gene

**Figure 1:**
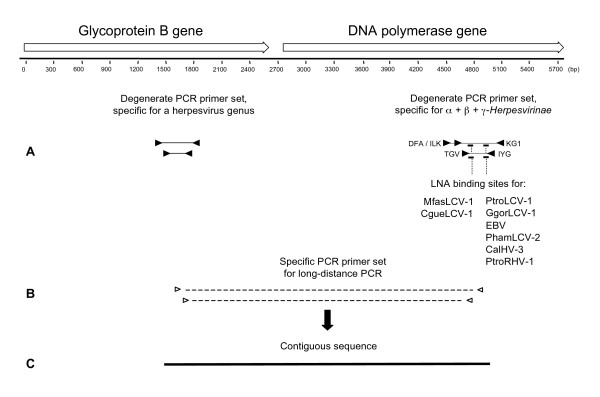
**LNA-based and bigenic amplification of beta- and gammaherpesviruses**. Schematic diagram of the analysis strategy. **(A, right) **Initially, panherpes nested PCR with deg/dI primers (black triangles) is performed for amplification of DPOL sequences. In the first round, primers for amplification of 710 bp and 480 bp are used simultaneously, either in the absence or presence of LNA. The binding regions of the LNA are present in the amplified sequences of both the first and the second PCR round, and represented by short thick lines. The targeted viruses are indicated. **(A, left) **The binding regions of genus-specific deg/dI gB-primers are indicated by black triangles. Amplimers of the first and the second PCR round are 320 bp and 250 bp, respectively. **(B) **After both gB and DPOL sequences were determined, long-distance nested PCR (dashed lines) was performed with specific primers (open triangles) binding to gB (sense) and DPOL (antisense). **(C) **A final contiguous sequence of approximately 3.5 kbp was obtained (solid line).

Pan-herpes PCR for amplification of 160 bp – 181 bp (without primer binding sites) of the DPOL gene [2] was carried out in a nested format with the degenerate and deoxyinosine-containing (deg/dI) primers DFA, ILK and KG1 in the first PCR round and TGV and IYG in the second round (Figure 1) as described previously [6]. For LNA evaluation, the second round was carried out as real-time PCR as described below.

### Pan-herpes PCR in the presence of LNA

LNA-substituted oligonucleotides (LNA) (TIB MOLBIOL GmbH, Berlin, Germany) were used to specifically inhibit the amplification of primate lymphocryptovirus (LCV) DPOL sequences, namely those of Pan troglodytes lymphocryptovirus 1 (PtroLCV-1), Gorilla gorilla lymphocryptovirus 1 (GgorLCV-1), Epstein-Barr virus (EBV), Macaca fascicularis lymphocryptovirus 1 (MfasLCV-1), Colobus guereza lymphocryptovirus 1 (CgueLCV-1), Papio hamadryas lymphocryptovirus 2 (PhamLCV-2) [7] and Callitrichine herpesvirus 3 (CalHV-3) [17]. An additional LNA was used to inhibit DPOL amplification of Pan troglodytes rhadinovirus 1 (PtroRHV-1; this study). To prevent the LNAs to function as PCR primers, an NH_2_-residue was added at their 3'-end. All LNAs are listed in Table 2.

**Table 2:** LNA sequences

LNA (Name)	LNA (Sequence)^$^	Target DPOL sequence	T_m _of DNA-oligomer (°C)^#^	T_m _of LNA-oligomer (°C)^#^	ΔT_m _(°C)
LNA-PtroLCV1	5'- +a+tg+a+cg+cg+t+ag+c+c+g --NH_2_	PtroLCV-1	55	82	27
LNA-GgorLCV1	5'- +at+a+acgcgt+a+gcc+g+accc --NH_2_	GgorLCV-1	51	77	26
LNA-EBV	5'- g+atg+act+cgaag+ctgg+ccct --NH_2_	EBV	63	72	9
LNA-MfasLCV1	5'- g+tggcc+a+acggcc+tc --NH_2_	MfasLCV-1 and Cgue LCV-1	60	69	9
LNA-PhamLCV2	5'- asg+ac+acgc+a+accgg --NH_2_	PhamLCV-2	59	67	8
LNA-CalHV3	5'- ac+tcgcag+ta+tacca+tccg --NH_2_	CalHV-3	54	69	14
LNA-PtroRHV1	5'- +a+acc+t+tg+a+a+tc+tggcg+tc -- NH_2_	PtroRHV-1	56	72	15

^$^All bases in LNA conformation are preceeded by +

^# ^T_m _calculated with the Exiqon Tm prediction algorithm.

**Figure 2:**
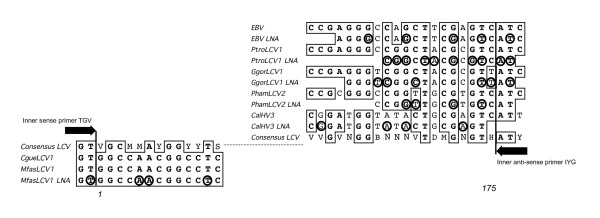
**Alignment of LNA-substituted oligonucleotides**. The LNAs, used in this study for specific inhibition of LCV amplification, were multiply aligned with their target sequences. The LNA-substituted bases are highlighted by circles. Identical nucleotides are boxed. For comparison, an LCV consensus sequence is shown, which was derived from all published LCV DPOL sequences. The bases 1 and 175 are the first and the last base of the sequence, which is amplified from LCV with pan-herpes DPOL primers (Bases 154668 and 154494 of the EBV B 95–8 genome [Acc. V01555], respectively). Only the 5'- and the 3'-ends are shown, the central part of the consensus sequence is represented by the dotted line. The 3'- ends of the binding regions of the inner consensus primers TGV (sense) and IYG (antisense) are indicated.

LNA-PtroLCV1, LNA-GgorLCV1, LNA-EBV, LNA-PhamLCV2, LNA-CalHV3 and LNA-PtroRHV1 specifically target the centre of the PtroLCV-1, EBV, PhamLCV-2, CalHV-3 and PtroRHV-1 DPOL sequences, respectively, which are amplified in the first round, and the 3'-end of the DPOL amplimers which are amplified in the second round of the pan-herpes DPOL PCR. With the exceptions of LNA-CalHV3 and LNA-PtroRHV1, the LNAs overlap the binding region of the inner anti-sense primer (IYG) by 2–3 bp (Figures 1 and 2).

LNA-MfasLCV1 specifically targets the 5'-end of both the MfasLCV-1 and the CgueLCV-1 sequence of the second round of the pan-herpes DPOL PCR. It overlaps with the binding region of the inner sense primer (TGV) by 2 bases (Figures 1 and 2).

All LNAs were added to the PCR reaction mixes of both the first and the second round of the pan-herpes DPOL PCR in the same concentration as the PCR primers (1 μM).

### Consensus-PCR with specificity for the glycoprotein B gene and for the major DNA binding protein gene of cytomegaloviruses

For the amplification of the gB gene, deg/dI primers were used in a nested format (Table 3). The primers were deduced from the gB genes of Equine herpesvirus 2 (primer set RH-gB), Epstein-Barr Virus (set LC-gB) and Human Cytomegalovirus (set CM-gB) and used for amplification of members of the genera *Rhadinovirus, Lymphocryptovirus *and *Cytomegalovirus*, respectively. They were degenerated and substituted with deoxyinosine at their 3'-end. Their binding region is depicted in Figure 1. PCR was carried out as described for the DPOL gene, with an annealing temperature of 45°C.

**Table 3:** Primers for amplification of the glycoprotein B gene

Primer set	Name of primer	PCR round	Sequence 5'- 3'	Product length ^$ ^(bp)
RH-gB	2759s	1	CCTCCCAGGTTCARTWYGCMTAYGA	700
	2762as		CCGTTGAGGTTCTGAGTGTARTARTTRTAYTC	
	2760s	2	AAGATCAACCCCAC(N/I^#^)AG(N/I)GT(N/I)ATG	500
	2761as		GTGTAGTAGTTGTACTCCCTRAACAT(N/I)GTYTC	
				
LC-gB	2753s	1	CCATCCAGATCCARTWYGC(N/I)TAYGA	650
	2756as		GATGTTCTGCGCCTRRWARTTRTA	
	2754s	2	TGGCTGCCAAGCG(N/I)(N/I)T(N/I)GG(N/I)GA	460
	2755as		GATGTTCTGCGCCTGRWARTTRTAYTC	
				
CM-gB	2743s	1	CGCAAATCGCAGA(N/I)KC(N/I)TGGTG	330
	2746as		TGGTTGCCCAACAG(N/I)ATYTCRTT	
	2744s	2	TTCAAGGAACTCAGYAARAT(N/I)AAYCC	250
	2745as		CGTTGTCCTC(N/I)CC(N/I)ARYTG(N/I)CC	
				
CM-MDBP	3730s	1	TGTGGCTTCTCATGCTTvCA[n/i]TT[n/i]TG	560
	3730as		GTTGAGGCTCCG[n/i]TCsAC[n/i]CC	
	3731s	2	CTATCTCGAGCATCG[n/i]TTyCAyAAC	350
	3731as		AAAAGTACCCAATCTG[n/i]CCrAAsTG	

^# ^I = Inosine

^$ ^approximate values

For the major DNA binding protein (MDBP) gene amplification of members of the genus *Cytomegalovirus*, nested consensus PCR was carried out with deg/dI primers, which were deduced from the MDBP gene of Cercopithecine herpesvirus 8 (CeHV-8) (Table 3). The PCR was carried out as described for the DPOL gene, with an annealing temperature of 46°C.

### PCR under less stringent conditions

Samples without amplification product in the panherpes DPOL PCR and in all gB PCRs were reanalysed under more relaxed conditions i.e. the ramp time between the annealing step and the extension step was prolonged 50-fold. In addition, the polymerase was only partially activated before cycling (2 min at 90°C), and the number of cycles was increased from 45 to 50.

### Long-distance PCR

LD-PCR was performed with the TaKaRa-Ex PCR system (Takara Bio Inc., Japan) or the Long-template PCR system (Roche, Switzerland) according to the manufacturer's instructions, and amplimers were obtained by nested PCR. For the second round, a one μl aliquot of the first round was used as template.

### Specific amplification of DPOL sequences from lymphocryptoviruses

From EBV, PtroLCV-1, GgorLCV-1 and CalHV-3, segments of the respective DPOL genes (approximately 1 kbp) were amplified (primers not listed). The amplimers span the entire binding region of the deg/dI pan-herpes DPOL primers, and were used in dilution series to test the LNA efficiency in the pan-herpes DPOL PCR.

### Real-time PCR

For the quantitative evaluation of LNA efficiency, the second round of pan-herpes DPOL PCR was performed as real-time PCR. The PCR mix was made up to a volume of 25 *μ*l containing 1.5 *μ*l of the first round reaction product, 1 × PCR buffer, 2 mM MgCl^2^, 0.2 mM (each) of dATP, dCTP, dGTP and dTTP (Fermentas, St. Leon-Rot, Germany, respectively), 2 U of AmpliTaq Gold DNA polymerase (Applied Biosystems, Foster City, CA, USA), 1 *μ*M (each) of the forward and reverse primers, 1.0 *μ*M SYBR Green I and 1.0 *μ*M ROX as a passive reference. LNAs were added to the PCR reaction mix in a concentration of 1 *μ*M. The reactions were carried out in 8-tube-strips (ABgene, Epsom-Surrey, UK) using an ABI Prism 7500 Sequence Detector (Applied Biosystems, Foster City, CA, USA).

### Sequence analysis and phylogenetic tree construction

PCR product purification, direct sequencing with dye terminator chemistry as well as nucleotide and amino acid sequence analysis were performed as described [18]. Sequence files were assembled with the Seqman module of the Lasergene software (GATC, Konstanz, Germany). BLAST searches were performed using the NCBI database. ORF prediction and calculation of identity values were performed with the program MacVector (Version 8.0). Multiple sequence alignments were performed with the clustalW module of MacVector. For phylogenetic tree construction, a multiple alignment of concatenated 1100 amino acids (aa) was analysed with the neighbor-joining method (MacVector). In addition, the alignment was analysed with the program Tree-Puzzle (Version 5.0).

### Tentative nomenclature of novel herpesviruses

For the purpose of this study, the novel viruses were named trinomially: The first 2 words designate the name of the host species, while the third word designates the tentative assignment of the novel virus to a herpesvirus genus within the *Herpesviridae*. The numbering was done according to the chronological order of discovery. Example: Macaca fascicularis rhadinovirus 1.

Abbreviations use the first letter of the generic host name and the first three letters of the specific host name, followed by the abbreviation of the viral genus. Example: **M**acaca **fas**cicularis rhadinovirus **(RHV) **1, MfasRHV-1.

### Nucleotide sequence accession numbers

Accession numbers for sequences of published viruses are:*Betaherpesvirinae*: HCMV (cg, NC 001347); HHV-6A (cg, NC 001664); HHV-7 (cg, NC 001716); PtroCMV-1 (cg, NC 003521). *Gammaherpesvirinae*: CalHV-3 (cg, NC 004367); CeHV-15 = Rhesus LCV (cg, X00784); EBV (cg, NC 007605); PtroLCV-1 (AF534226); MfasLCV-1 (AF534221); CgueLCV-1 (AF534219); HHV-8 (cg, NC 003409); HVS = SaHV-2 (cg, NC 001350); RRV strain 17577 (cg, AF083501); RRV strain 26–95 (cg, AF210726).

The novel sequences reported here were deposited in GenBank under the following accession numbers: PtroRHV-1, acc. AY138585; PtroRHV-2, acc. EU085378; MfasRHV-1, acc. AY138583; MfasRHV-2, acc. EU085377; PhamLCV-2, acc. AF534229; PhamLCV-3, acc. EU11846; CgueCMV-1.1, acc. AY129397; CgueCMV-1.2, acc. EU11847.

## Results

### LNA-substituted oligonucleotides specifically inhibit the amplification of DPOL sequences in the pan-herpes DPOL PCR

Approximately 1 kbp of the PtroLCV-1, the GgorLCV-1, the EBV and the CalHV-3 DPOL gene were amplified with specific primers (not listed). These PCR fragments span the complete DPOL region targeted by pan-herpes consensus PCR (Figure 1). Serial ten-fold dilutions of these fragments, covering a range of 10^7 ^to 10^-1 ^copy numbers, were used as templates in the pan-herpes DPOL PCR, either in the presence or absence of perfectly matching LNAs.

**Figure 3:**
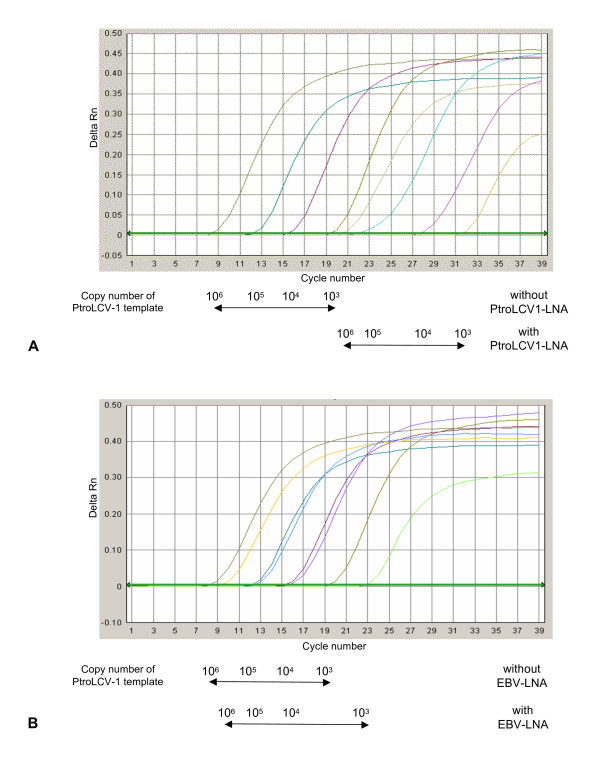
**Real-time PCR of PtroLCV-1 DPOL in the presence or absence of LNA**. Amplification curves are shown for pan-herpes DPOL PCR with a PtroLCV-1 DPOL amplimer (1 kbp) as template (10^3 ^– 10^6 ^copy numbers). Real-time PCR was carried out in the presence or absence of **(A) **LNA-PtroLCV1 or **(B) **LNA-EBV.

The amplification of PtroLCV-1 DPOL was severely impaired by LNA-PtroLCV1. In the presence of this LNA, a minimum of 1000 copies was needed to obtain an amplimer visible after gel-electrophoretic analysis (not shown). In the absence of LNA-PtroLCV1, 1–10 copies of PtroLCV-1 were still amplifiable. Similar results were achieved by repeating the second PCR round in the presence of SYBR green I in a real-time set-up. In the presence of LNA-PtroLCV1, the C_T_-values rose by 11–12 cycles (Figure 3a). From this data it was concluded that LNA-PtroLCV1 inhibited the amplification of PtroLCV-1 DPOL by a factor of approximately 1000.

A similar inhibition efficiency was seen when the GgorLCV-1 DPOL template was amplified in the presence of LNA-GgorLCV1 (factor approximately 1.000). The EBV DPOL template was even more effectively inhibited in the presence of LNA-EBV (factor >10.000) (data not shown).

Various LNA concentrations were tested for their inhibition efficiency. Concentrations of 4 μM, 2 μM, 1 μM and 0.5 μM of LNA-PtroLCV1 inhibited the amplification of PtroLCV-1 DPOL to a similar degree. A concentration of 0.25 mM resulted in a decreased inhibition (not shown). For the remainder of the experiments presented here a 1 μM of LNA was routinely used.

We next tested, whether LNAs exert their effect in a sequence-specific manner. For this purpose, EBV DPOL was amplified in the presence of the LNA-PtroLCV1 that contains 3 mismatches within the LNA binding region. Two of the mismatches were LNA-substituted (Figure 2). In the presence or absence of the LNA, a similar amount of amplimer was obtained in real-time PCR. Thus, the amplification of EBV DPOL was not inhibited by the LNA-PtroLCV1 (not shown). Conversely, the PtroLCV-1 template was tested with LNA-EBV, which also exhibits 3 mismatches within the LNA binding region. However, no mismatch was LNA-substituted (Figure 2). Using 10^3 ^template molecules, a slight inhibition (factor of <10) was observed. Higher template concentrations were not affected by the LNA-EBV as revealed by real-time PCR (Figure 3b).

Finally, we tested the impact of LNAs, which exhibit only one or two mismatches to a certain DPOL sequence in their binding region. LNAGgorLCV1 exhibits two mismatches to the PtroLCV-1 DPOL, and both were LNA-substituted (Figure 2). With LNA-GgorLCV1, the inhibitory effect on amplification of the PtroLCV-1 DPOL sequence was 10-fold lower than on the exactly matching GgorLCV-1 DPOL sequence. In the case of the LNA-PtroLCV1, the one mismatch to the GgorLCV-1 sequence was not LNA-substituted (Figure 2), and LNA-PtroLCV1 inhibited the amplification of GgorLCV-1 DPOL to the same extent as the amplification of the perfectly matching PtroLCV-1 template. Thus LNA-PtroLCV1 did not discriminate between these templates (data not shown).

### Selective amplification of herpesvirus DPOL templates from template mixtures using pan-herpes PCR and LNA-substituted oligonucleotides

Three mixtures of the 1 kbp templates of the GgorLCV-1, EBV and CalHV-3 DPOL genes were prepared. In each of these, one template was present in a copy number representing its individual detection limit in the pan-herpes PCR. The other two templates were present in 10-fold excess over their individual detection limit. Pan-herpes PCR was set up in the presence of two LNAs targeting the two over-represented HV. In each LNA-supplemented PCR assay, the amplification of the two over-represented HV was inhibited and the single under-represented HV was amplified as revealed by sequencing (Table 4, assays A to C). The experiment was repeated, but with the two over-represented HV present in 100-fold excess. In the assays D and E, listed in Table 4, the under-represented GgorLCV-1 DPOL and EBV DPOL were selectively amplified, respectively. However, when CalHV-3 was under-represented, the 100-fold over-represented GgorLCV-1 DPOL was detected instead (Table 4, assay F).

**Table 4:** Amplification of an under-represented LCV species in the presence of two over-represented, closely related LCV species

		Viral templates added to the PCR reaction	Copy number of added template	Pan-herpes PCR detection limit (template copy number)	Ratio between added template concentration and the detection limit	LNA added	Expected virus detection	Detected virus
A	1	EBV	10.000	10.000	1	-		
	2	GgorLCV1	100	10	10	GgorLCV1	EBV	EBV
	3	CalHV3	10	1	10	CalHV3		
								
B	1	EBV	100.000	10.000	10	EBV		
	2	GgorLCV1	10	10	1	-	GgorLCV1	GgorLCV1
	3	CalHV3	10	1	10	CalHV3		
								
C	1	EBV	100.000	10.000	10	EBV		
	2	GgorLCV1	100	10	10	GgorLCV1	CalHV3	CalHV3
	3	CalHV3	1	1	1	-		
								
D	1	EBV	10.000	10.000	1	-		
	2	GgorLCV1	1000	10	100	GgorLCV1	EBV	EBV
	3	CalHV3	100	1	100	CalHV3		
								
E	1	EBV	1000.000	10.000	100	EBV		
	2	GgorLCV1	10	10	1	-	GgorLCV1	GgorLCV1
	3	CalHV3	100	1	100	CalHV3		
								
F	1	EBV	1000.000	1.000	100	EBV		
	2	GgorLCV1	1000	10	100	GgorLCV1	CalHV3	GgorLCV1
	3	CalHV3	1	1	1	-		

To further investigate the versatility of the LNA-supplemented panherpes PCR, four identical mixtures of four LCV templates were prepared. This time, template concentrations were chosen at which amplification was only partially inhibited by the LNA. Three LNAs were added to each of the four PCR reactions in four different combinations. From each mixture the expected sequence was amplified i.e. that sequence against which no corresponding LNA had been added (data not shown). It was concluded that at least four unknown herpesviruses might be selectively amplified from multi-infected samples using a panel of LNAs.

### Discovery of rhadinoviruses in lymphocryptovirus-positive samples of chimpanzees

The LNAs, which were successfully used in dissecting artificial LCV template mixtures, were now used for the analysis of herpesvirus-positive blood and tissue samples of primates. Samples of two chimpanzees ("Noah" and "Leo"; *Pan troglodytes verus*), which lived in the Taï National Park of Côte d'Ivoire and died from anthrax disease [19], were analysed with pan-herpes DPOL PCR resulting in the detection of PtroLCV-1 [7]. To unravel the simultaneous presence of other herpesviruses, the pan-herpes DPOL PCR was carried out in the presence of LNA-PtroLCV1, using spleen samples of Noah and Leo. As a control, the PCR was carried out without LNA. While in the control reaction PtroLCV-1 DPOL was amplified, the presence of the LNA resulted in the amplification of a novel RHV DPOL sequence. The virus, from which this sequence originated, was tentatively named PtroRHV-1.

To amplify a gB sequence of PtroRHV-1, the gB primer set RH-gB was then applied to several samples from Noah, Leo and chimpanzees of the same group. In a sample of the chimpanzee "Gargantuan", a RHV gB sequence was detected. It could be connected to the PtroRHV-1 DPOL sequence by LD-PCR, and therefore originated from PtroRHV-1. A 3.4 kbp sequence was finally compiled spanning the 3'-end of the gB gene (approximately 1 kb) and the 5'-end of the DPOL gene (approximately 2.2 kb) of PtroRHV-1 (Figure 1).

In the spleen samples of Noah and Leo, a different RHV gB sequence was detected, as indicated by an identity percentage of only 70% to the PtroRHV-1 gB sequence. The virus, from which this gB sequence originated, was tentatively named PtroRHV-2.

**Figure 4:**
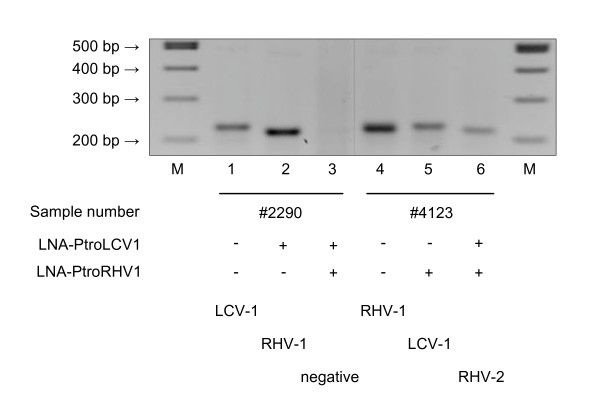
**Pan-herpes DPOL PCR of chimpanzee samples in the presence or absence of LNA**. Pan-herpes DPOL PCR was carried out on aliquots of chimpanzee Leo's spleen (#2290; #4123) in the absence (-) or presence (+) of LNA-PtroLCV1 and/or LNA-PtroRHV1. The electropherogram is shown. Below, the amplified DPOL sequences are indicated. Marker: 100 bpladder (lanes 1 and 8).

To amplify the DPOL sequence of PtroRHV-2, two aliquots of Leo's spleen, originating from different regions within the organ, were subjected to pan-herpes DPOL PCR. This time two LNAs were used (LNA-PtroLCV1 and LNA-PtroRHV1; Table 2 and Figure 1) to simultaneously inhibit the amplification of PtroLCV-1 and PtroRHV-1 DPOL and thus to be able to detect PtroRHV-2 DPOL. In aliquot 1 of Leo's spleen, PtroLCV-1 was detected in the absence of the LNAs. By including LNA-PtroLCV1, PtroRHV-1 was detected. Using both LNAs, nothing was detected. Therefore, the sample did apparently not contain PtroRHV-2 in a copy number sufficient for pan-herpes PCR (Figure 4). However, the dual-inhibition approach proved successful with the second spleen aliquot. Using both LNAs, a second RHV DPOL sequence was discovered (Figure 4). It revealed a percentage of identity to PtroRHV-1 DPOL of 52%. This DPOL sequence could be connected to the PtroRHV-2 gB sequence by LD-PCR, and therefore originated from PtroRHV-2. A 3.4 kbp sequence of PtroRHV-2 was finally compiled (Figure 1).

In a pair-wise nucleic acid sequence comparison, the 3.4 kbp sequence of PtroRHV-1 was found to be 99% identical in its 3'-terminus to a 1 kbp DPOL sequence detected in a captive *Pan troglodytes troglodytes *[20]. The 3.4 kbp sequence of PtroRHV-2 was 98% identical in its 3'-terminus to a 1.1 16 kbp DPOL sequence detected in three wild-caught *Pan troglodytes troglodytes*, one from Gabon and two from Cameroon [21]. No close matches were found in Genbank for the 5'-parts (2.4 kbp) of the PtroRHV-1 and PtroRHV-2 sequences, spanning a part of the DPOL and the gB gene. Since both originated from *Pan troglodytes verus *(Côte d'Ivoire), they were regarded as originating from hitherto unknown *P. tr. verus *RHV, closely related to *P. tr. troglodytes *RHV.

### Discovery of rhadinoviruses in LCV-positive samples of cynomolgus monkeys

Blood and organ samples of 18 cynomolgus monkeys (*Macaca fascicularis*) from the colony of the German Primate Centre were analysed with panherpes DPOL PCR. In 21 out of 35 samples, amplimers of Macaca fascicularis lymphocryptovirus 1 (MfasLCV-1) [7] were obtained (data not shown). In one blood sample, a rhadinovirus DPOL sequence was found and the virus tentatively named Macaca fascicularis rhadinovirus 1 (MfasRHV-1). Re-inspection of the individuals with the gB primer set RH-gB revealed two RHV gB sequences (RHV1-gB and RHV2-gB). They had only 72% nucleotide sequence identity to each other. LD-PCR revealed that the RHV1-gB sequence originated from the same virus genome as the MfasRHV-1 DPOL sequence. A final gB to DPOL sequence (3.4 kbp) of MfasRHV-1 was compiled.

The virus from which the RHV2-gB sequence originated was tentatively named MfasRHV-2. To amplify a DPOL sequence of MfasRHV-2, the panherpes DPOL PCR was carried out in the presence of LNA-MfasLCV1. As a control, the PCR was carried out without LNA. While MfasLCV-1 DPOL was amplified in the control reaction, the presence of the LNA resulted in the amplification of a second RHV DPOL sequence. This could be connected to the MfasRHV-2 gB sequence with LD-PCR, resulting in a final MfasRHV-2 gB to DPOL sequence of 3.4 kbp.

The MfasRHV-1 DPOL sequence was 95% identical to that of the retroperitoneal fibromatosis virus, a RHV detected in *Macaca mulatta *[22]. The 3'-end of the MfasRHV-2 sequence was 98% identical to a RHV sequence (475 bp), which had been detected in the USA in *M. fascicularis *originating from Indonesia (Genbank accession AF159032) [23]. Pairwise comparison of the complete gB to DPOL sequence of MfasRHV-1 with the corresponding sequences of (i) MfasRHV-2, (ii) rhesus monkey rhadinovirus and (iii) Human herpesvirus 8 revealed identities of 65%, 67% and 64%, respectively.

### Discovery of a third lymphocryptovirus species in an LCV-positive sample of a baboon

Two LCV of baboons (*Papio hamadryas*) are presently known, Papio hamadryas lymphocryptovirus 1 (PhamLCV-1 = Herpesvirus papio = *Cercopithecine herpesvirus 12*) [24] and Papio hamadryas lymphocryptovirus 2 (PhamLCV-2) [7]. While several genomic regions of PhamLCV-1 had been already determined, including the complete gB gene [25], only a short partial DPOL sequence of PhamLCV-2 had been described [7]. Therefore, we inspected five PhamLCV-2-positive *P. hamadryas *with the LC-gB primer set. Two different gB sequences were detected.

The first could be connected to the PhamLCV-2 DPOL sequence by LD-PCR, and a final PhamLCV-2 sequence of 3.2 kbp was obtained. The second gB sequence differed slightly from the corresponding gB sequences of PhamLCV-1 and PhamLCV-2 (90% and 95% identity, respectively). The virus from which this gB sequence originated was tentatively named PhamLCV-3.

To amplify a DPOL sequence of PhamLCV-3, the panherpes DPOL PCR was performed with and without LNA-PhamLCV2. In the control reaction PhamLCV-2 DPOL was amplified, while the presence of the LNA resulted in the amplification of a different LCV DPOL sequence (85% identity). This putative PhamLCV-3 sequence could be connected with the PhamLCV-3 gB sequence by LD-PCR, resulting in a final PhamLCV-3 gB to DPOL sequence of 3.3 kbp.

An alignment of the novel PhamLCV-3 sequence with LNA-PhamLCV2 revealed three mismatches. In addition, one of the mismatching bases within the LNA-PhamLCV2 was LNA-substituted. These features most probably prevented the targeting of PhamLCV-3 DPOL by LNA-PhamLCV2.

The three LCV of *P. hamadryas *were compared on the basis of gB sequences (a longer DPOL sequence was not available for PhamLCV-1). Identity percentages of 89% (LCV-3 versus LCV-1) and 93% (LCV-3 versus LCV-2) were obtained.

### Discovery of cytomegaloviruses in LCV-positive samples of a black-and-white colobus

Blood, spleen, brain, kidney, bone marrow, stomach and mucosa of the mouth of a black-and-white colobus (*Colobus guereza*) from the Berlin zoological gardens, which died of a disease of unclear etiology, were analysed with pan-herpes DPOL PCR. In 6/7 samples, the lymphocryptovirus CgueLCV-1 [7] was found. In the kidney, a novel cytomegalovirus was discovered and tentatively named Colobus guereza cytomegalovirus 1 (CgueCMV-1).

**Figure 5:**
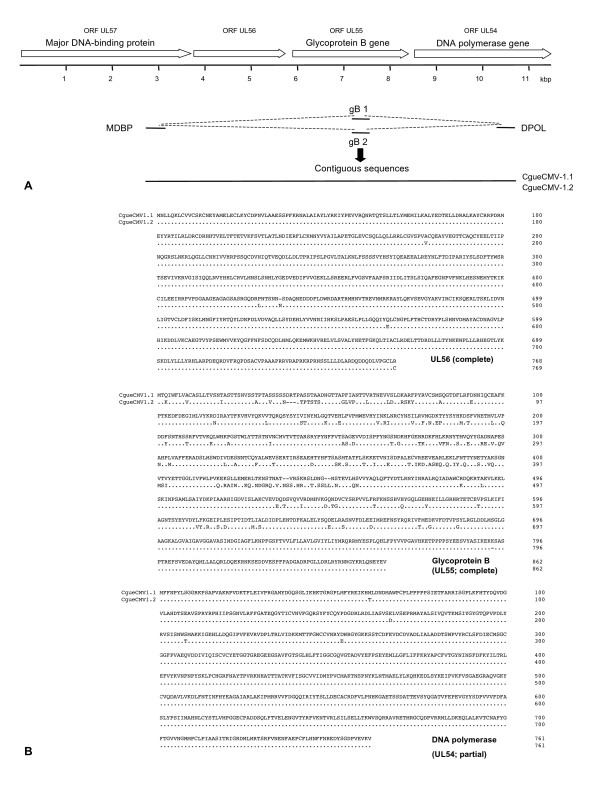
**Amplification of an 8 kbp locus of CgueCMV-1.1 and CgueCMV-1.2**. At the top of the figure, the betaherpesvirus ORFs UL57 (MDBP) to UL54 (DPOL) are depicted by open arrows. **(A) **The partial sequences of the ORFs UL57, UL55 and UL54, obtained through PCR with deg/dI primers, are depicted by thin solid lines, and the type of gB sequence (gB1 or gB2) is indicated. LD amplimers are depicted by dashed lines. The solid line represents the final 8 kbp contiguous sequences of CgueCMV-1.1 and CgueCMV-1.2. **(B) **Pairwise alignments of the UL56 and gB proteins and the partial DPOL proteins of CgueCMV-1.1 and CgueCMV-1.2 are shown. Dots represent identical amino acids, dashes indicate regions of non-colinearity.

Inspection of all samples with the gB primer set CM-gB revealed two distinct gB sequences. One was found in the kidney and brain and the other in liver and mucosa (CMV-1 and CMV-2, respectively). They had a nucleotide sequence identity of 82%. With LD-PCR, the CMV-1 gB sequence and the CgueCMV-1 DPOL sequence could be connected (Figure 5A).

The virus, from which the CMV-2 gB sequence was derived, was tentatively named CgueCMV-2. To amplify the missing DPOL sequence of CgueCMV-2, the CgueCMV-2-positive samples were subjected to the panherpes DPOL PCR in the presence of the LNA-MfasLCV1 (Table 3 and Figure 2). In the control reaction without LNA CgueLCV-1 DPOL was amplified, while the presence of the LNA-MfasLCV1 surprisingly resulted in the amplification of CgueCMV-1 DPOL with 100% identity. Because no other CMV sequence was found, we speculated that CgueCMV-2 might differ from CgueCMV-1 only in the gB gene but not in the DPOL gene. This was indeed the case, since we could connect the CgueCMV-2 gB sequence with the CgueCMV-1 DPOL sequence by LD-PCR (Figures 1 and 5A).

**Figure 6:**
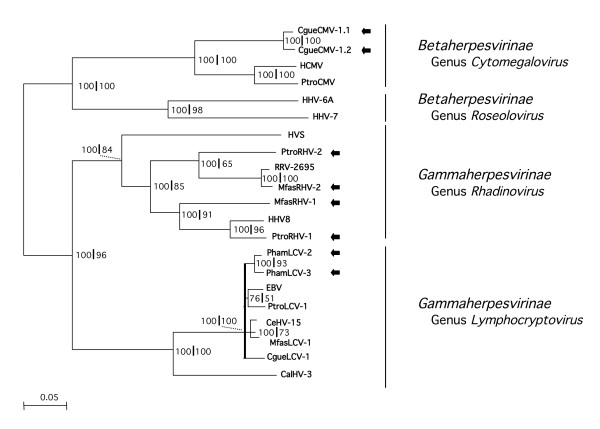
**Phylogenetic analysis of the novel primate herpesviruses**. A phylogenetic tree was constructed using the amino acid (aa) sequences encoded by the gB-DPOL segments of the novel primate herpesviruses and of known human and non-human primate herpesviruses, available in GenBank. A multiple alignment of concatenated 1100 aa was analysed with the neighbor-joining method. A midpoint-rooted phylogram is shown. The branch length is proportional to evolutionary distance (scale bar). Results of bootstrap analysis (100-fold) are indicated at the nodes of the tree, to the left of the first vertical divider. In addition, the alignment was analysed with Tree-Puzzle 5.0. Support values, estimated by the quartet puzzling (QP) tree search and expressing the QP reliability in percent, are indicated at the nodes of the tree to the left of the vertical divider. Nodes with values below 70% in both analyses were depicted as pat of a multifurcation (black bar). Viruses, which are entirely novel or viruses for which additional sequence information was generated, are highlighted with black arrows. Herpesvirus genera and families are indicated. Full names of known viruses and their nucleotide sequence accession numbers are listed in the *Methods *section.

Pairwise comparison of both CgueCMV nucleotide sequences revealed a difference on the nucleotide level of 11% in the gB gene and only 2% in the DPOL gene. Therefore, they were regarded as variants of the same viral species and renamed CgueCMV-1.1 and CgueCMV-1.2. To evaluate, (i) how broad the differences are between the complete gB genes of both CgueCMV- 1.1 and CgueCMV-1.2 and (ii) whether the conserved ORFs upstream of the gB gene reveal extensive amino acid variations, we amplified with degenerate primers and sequenced a part of the gene (ORF UL57) encoding for the MDBP (Figure 5A). This sequence was connected with both the gB sequences of CgueCMV-1.1 and CgueCMV-1.2 by LD-PCR (Figure 5A). For both viruses, a final sequence of about 8 kb was determined, encoding a part of the ORF UL57 (MDBP), the complete ORFs UL56 and UL55 (gB), and two thirds of the ORF UL54 (DPOL) (Figure 6B). The nucleotide and amino acid sequence differences of CgueCMV-1.1 and CgueCMV-1.2 were 0.6% and 0.5% (UL56), 20% and 8% (UL55) and 1% and 0.1% (UL54), respectively.

### Phylogenetic analysis of the novel beta- and gammaherpesviruses

A phylogenetic tree was constructed with concatenated aa sequences of gB and DPOL. It is the first comprehensive tree of primate beta- and ammaherpesviruses based on gB and DPOL sequences of more than 1000 aa. Nearly all viruses branched with a probability of 70–100%.

Both the *P. troglodytes *rhadinoviruses (PtroRHV-1 and PtroRHV-2) and the *M. fascicularis *rhadinoviruses (MfasRHV-1 and MfasRHV-2) strongly confirmed the concept of two distinct primate rhadinovirus lineages published earlier [26]. While PtroRHV-1 and MfasRHV-1 appeared as members of the RHV1 group (HHV8-like), PtroRHV-2 and MfasRHV-2 belonged to the RHV2 group of which the *M. mulatta *rhadinovirus RRV is the best-characterized member. The baboon lymphocryptoviruses PhamLCV-2 and PhamLCV-3 were closely related to each other and to PhamLCV-1. They clustered in the group of *Old World *primate lymphocryptoviruses, of which EBV is the prominent member. The colobus betaherpesviruses CgueCMV-1.1 and 1.2 appeared as closely related members of the genus *Cytomegalovirus *(Figure 6).

## Discussion

This is the first report describing the differential amplification of virus sequences by the aid of LNA-substituted oligonucleotides. The LNA inhibited the amplification of specific, perfectly matching HV DPOL sequences and enabled the discovery of other, unknown HV DPOL sequences. The versatility of this approach was demonstrated by amplification of six novel primate herpesvirus DPOL sequences from multi-infected samples. In these experiments, LCV DPOL and RHV DPOL amplification was inhibited by LNA addition. Furthermore, we successfully used CMV-specific LNAs to inhibit the amplification of human CMV DPOL and gB genes and an LCV-specific LNA to inhibit the amplification of ORF11 of LCV (Sandra Prepens, Merlin Deckers, Katja Spieß and Bernhard Ehlers, unpublished data). We concluded that the amplification of every HV gene might be accessible to inhibition by LNAs.

LNA-containing oligonucleotides exhibit varying efficacies and specificities with regards to their inhibitory potential. Several factors may account for these differences: The primary sequence, the number of introduced LNA bases and their position within the oligonucleotide, and the secondary structures of the templates. We positioned most LNAs at the 3'- end of the second round amplification product, as this is the region of the highest sequence diversity among the amplification products of the panherpes DPOL PCR. However, we accepted possible interferences by secondary structures in some PCR templates. This may account for the fact that the LNA-EBV was 10 times more effective than LNA-PtroLCV1 and that 2 other LNAs had only partial inhibitory activity (data not shown).

The positioning of the LNA-binding region to the comparatively variable 3'-end of the second round amplimer allowed the amplification of HV DPOL sequences that differ only slightly from the LNA-targeted HV DPOL sequence. In artificial template mixtures, closely related HV sequences with only 2 to 3 mismatches in the LNA-binding site could be selectively dissected by LNA addition. Furthermore, the novel PhamLCV-3 DPOL sequence was found in an organ sample, which was also positive for PhamLCV-2. Both sequences differ by only 3 bases in the LNA-binding site. The LNA selectivity was likely supported by insertion of LNA bases exactly at mismatch positions as illustrated by the higher specificity of the LNA-GgorLCV1 (LNA substitutions at both mismatch positions; Figure 2) compared to that of the LNA-PtroLCV1 (no LNA substitution at the mismatch position; Figure 2). Similar positional effects were reported using LNA-substituted oligonucleotides as real-time PCR probes [15,27]. Of course the selective placement of LNA bases at mismatch positions cannot be carried out for unknown sequences. Here, it is left to chance.

For inhibition of MfasLCV-1 and CgueLCV-1 amplification, we used an LNA targeting the 5'-end of the amplification product. This region is more conserved than the 3'-end and does not allow for discrimination of closely related sequences. However, non-LCV sequences (RHV or CMV) were to be amplified in both cases, and therefore a single, more conserved LNA was suitable in both experiments.

The LNA technique was supplemented with amplification of the gB gene, using different sets of genus-specific primers. With these, the amplification of 8 novel primate gB sequences was achieved. Besides their general usefulness, both detection approaches have specific constraints. Different sets of degenerate, genus-specific gB primers can in principle amplify as many herpesvirus gB sequences from a single sample as herpesvirus genera for a certain host species exist. However, this can become laborious as exemplified by primate herpesviruses. Two alpha-, two beta- and two gammaherpesvirus genera are known requiring at least 6 nested sets of gB primers. Furthermore, viruses of quite similar sequence will exhibit an identity of close to 100% in the binding regions of the consensus primers. Therefore, they cannot be differentiated from the same sample by gB PCR (without LNA addition).

In contrast to gB PCR, pan-herpes DPOL PCR in the presence of a sequence-specific LNA could differentiate between very similar DPOL genes from the same genus. An artificial mixture of four similar LCV templates could be dissected by the addition of three LNAs, and two very similar LCVs were found in a baboon spleen sample (PhamLCV-2 without LNA; PhamLCV- 3 with LNA). Furthermore, simultaneous addition of two LNAs resulted in the exclusion of two viruses (PtroRHV-1 and PtroLCV-1) and enabled the amplification of a third virus (PtroRHV-2) from a single organ sample (Figure 4). Based on this data we speculate that at least four herpesvirus species might be discovered in a single sample. However, when viruses like CgueCMV-1.1 and CgueCMV-1.2 are simultaneously present which do not differ in their DPOL but only in their gB genes, differentiation would only be possible by (LNA-supplemented) amplification of the gB gene.

For robust phylogenetic tree construction, the parallel targeting of two conserved genes like DPOL and gB is superior to approaches used previously [11,7,28]. In those reports, the initial short DPOL consensus sequence of <200 bp was extended in upstream direction by about 300 bp with 2 rounds of semi-nested, semi-specific PCR. This had resulted in a contiguous sequence of approximately 480 bp. Although this had improved the probability of phylogenetic trees, the whole approach still yielded limited additional sequence information. Here we present amplification of >3 kb sequences which encode for approximately 350 aa of gB and 750 aa of DPOL. Such data allows for the construction of phylogenetic trees of significantly higher probability as exemplified by the tree of primate beta- and gammaherpesviruses presented here (Fig. 6).

The colobus monkey was infected with two different strains of cytomegalovirus in many organs. Presently, we do not know whether this double CMV infection contributed to the death of the animal. In humans, the simultaneous infection with different herpesviruses is not uncommon and was linked to enhanced pathogenicity and disease impact [29-32]. Moreover, mixed gB genotypes of human CMV (HCMV) were found in immunocompromised patients [33], and specific HCMV gB genotypes were associated with several human diseases [reviewed by [34]]. Therefore, technology for differentiating unknown viruses or unknown variants of recognized viruses in clinical specimens is needed, and this requirement adds to the importance of the presented methodology.

Very little information is available on the spectrum of viruses in primates living in their natural habitats [35]. The LNA methodology, presented here, may become an effective tool to comprehensively screen primates for unknown pathogens, in particular those with zoonotic potential.

Finally we predict that this novel technical approach is in principle applicable to dissect mixed infections with viruses from every viral family.

## Abbreviations

CalHV-3 Callitrichine herpesvirus 3

CeHV-8 Cercopithecine herpesvirus 8

CeHV-15 Cercopithecine herpesvirus 15

CgueCMV-1.1 Colobus guereza cytomegalovirus 1.1

CgueCMV-1.2 Colobus guereza cytomegalovirus 1.2

CgueLCV-1 Colobus guereza lymphocryptovirus 1

EBV Epstein-Barr virus

GgorLCV-1 Gorilla gorilla lymphocryptovirus 1

HCMV Human cytomegalovirus

HHV-6A Human herpesvirus 6A

HHV-7 Human herpesvirus 7

HHV-8 Human herpesvirus 8

HVS Herpesvirus saimiri

MfasLCV-1 Macaca fascicularis lymphocryptovirus 1

MfasRHV-1 Macaca fascicularis rhadinovirus 1

MfasRHV-2 Macaca fascicularis rhadinovirus 2

PhamLCV-1 Papio hamadryas lymphocryptovirus 1

PhamLCV-2 Papio hamadryas lymphocryptovirus 2

PhamLCV-3 Papio hamadryas lymphocryptovirus 3

PLHV-1 Porcine lymphotropic herpesvirus 1

PtroCMV-1 Pan troglodytes cytomegalovirus 1

PtroLCV-1 Pan troglodytes lymphocryptovirus 1

PtroRHV-1 Pan troglodytes rhadinovirus 1

PtroRHV-2 Pan troglodytes rhadinovirus 2

RRV Rhesus monkey rhadinovirus

SaHV-2 Saimiriine herpesvirus 2

## Competing interests

The author(s) declare that they have no competing interests.
